# Extraction and processing of intensive care chart data from a patient data management system

**DOI:** 10.3389/fdgth.2026.1747227

**Published:** 2026-03-19

**Authors:** Nikolas B. Schrader, Burkhard Meißner, Paul Fischer, Daniel Röder, Maximilian Ertl, Patrick Meybohm, Benedikt Schmid

**Affiliations:** 1 Department of Anaesthesiology, Intensive Care, Emergency and Pain Medicine, University Hospital Würzburg, Würzburg, Germany; 2 Service Center Medical Informatics, University Hospital Würzburg, Würzburg, Germany

**Keywords:** anaesthesia, extract transform and load (ETL), intensive care medicine, patient data management system (PDMS), Python (programming language), SQL (structured query language)

## Abstract

**Background:**

Routine clinical data captured in Patient Data Management Systems (PDMS) in intensive care and perioperative settings are an invaluable resource for clinical research. However, the proprietary, fragmented, and transaction-oriented architecture of many systems severely limits secondary data use and requires extensive Extract, Transform, and Load (ETL) processing.

**Methods:**

We developed a modular, Python-based ETL framework that enables flexible, domain-specific extraction of high-frequency, multimodal PDMS data. The system provides reusable components for data retrieval, preprocessing, harmonization, and de-identification, allowing extraction methods to be adapted or extended without modifying the core architecture. Each clinical domain is represented through dedicated Pydantic models enforcing consistent output schemas, type constraints, and automated plausibility checks. SQLAlchemy abstracts database access, while structured preprocessing logic resolves common documentation inconsistencies and transforms heterogeneous PDMS entries into standardized representations.

**Results:**

The framework produces reproducible, analysis-ready datasets through a transparent, auditable workflow. An integrated audit logger records extraction parameters, transformations, and derived fields, providing full traceability. Salted, irreversible pseudonymization is embedded directly into the pipeline, supporting compliance with the European General Data Protection Regulation (GDPR; German: Datenschutz-Grundverordnung, DSGVO) and Art. 27 of the Bayerisches Krankenhausgesetz (BayKrG). By encapsulating extraction logic in modular processing units with consistent validation and automated de-identification, the system replaces complex *ad hoc* queries with standardized, maintainable, and research-ready processes.

**Conclusion:**

The presented framework overcomes substantial technical and regulatory barriers to the secondary use of PDMS data by operationalizing a governance-first extraction pipeline. Its modular architecture encapsulates site-specific PDMS queries in a bounded adapter layer, while keeping validation, pseudonymization, and audit logging portable and reusable across domains and installations. By embedding domain-level validation models, irreversible pseudonymization, and structured auditing, the framework enables reproducible, governance-compliant access to high-frequency intensive care data. Rather than requiring immediate alignment to a common data model, it provides a pragmatic foundation on which semantic and syntactic interoperability can be added incrementally as requirements and resources evolve.

## 1 Introduction

The secondary use of routine clinical data from electronic health records (EHR) is an increasingly valuable resource for clinical research (1, 2), quality improvement (3), and the development of decision-support systems (4). Intensive care medicine (ICM) and perioperative settings, in particular, generate high-frequency, multimodal data captured within Patient Data Management Systems (PDMS). While these systems facilitate accurate, detailed and flexible clinical documentation, they are not designed to provide direct access for research purposes. Consequently, Extract, Transform and Load (ETL) processes for secondary data use face significant methodological and technical challenges (5). Different individual approaches to address these challenges have been published before (6, 7).

For clarity, this paper uses the following terminology consistently: *Electronic Health Record (EHR)* refers broadly to hospital-wide digital patient record systems; *Patient Data Management System (PDMS)* refers to specialised intensive care/perioperative documentation systems that generate high-frequency bedside data; and *clinical data warehouse (CDW)* refers to an analytics-oriented repository populated via ETL from operational systems.

Access to and utilization of EHR data is subject to a plethora of laws and regulations. In Germany, the secondary use of hospital routine data is governed by strict legal frameworks, most notably the European General Data Protection Regulation (GDPR; Datenschutz-Grundverordnung, DSGVO) and the Bayerisches Krankenhausgesetz (BayKrG). Together, these regulations establish the legal basis for the pseudonymized secondary use of routine hospital data for scientific purposes, provided that appropriate technical and organizational safeguards are implemented.

The Patient Data Management System (PDMS) Copra (Copra v6, COPRA System GmbH, Berlin, Germany) is a modular platform that has been widely implemented in intensive care and perioperative settings (8, 9). Its primary function is the continuous, real-time documentation of clinical information, supporting direct patient care and ensuring medico-legal traceability. The system acquires high-frequency physiological signals (e.g., heart rate, blood pressure, ventilatory parameters), discrete clinical events (e.g., medication administrations, laboratory results, procedures), as well as structured and unstructured data such as clinical scores and narrative notes.

These data points are stored within a relational database optimized for transactional integrity and operational performance, ensuring reliability and consistency of bedside documentation. However, this optimization is achieved at the expense of secondary data use. The underlying database schema is highly denormalized and fragmented, often featuring data redundancy and reliance on proprietary system-specific coding instead of standardized terminologies (e.g., LOINC, ATC). This leads to incomplete or inconsistently implemented data capturing logic. While these design choices safeguard clinical performance and medico-legal requirements, they present considerable barriers for analytical queries, research applications, and data interoperability.

However, the database remains technically accessible, allowing custom extractions via direct queries and tailored transformation pipelines. This accessibility provides a foundation for research-oriented workflows, even though substantial preprocessing and harmonization are required to access the data suitable for secondary use.

This paper presents a structured ETL methodology for data from our institutional PDMS. We discuss the inherent data pipeline hurdles and propose replicable solutions to make high-quality, analysis-ready data accessible to the wider research community.

## 2 Methods

### 2.1 High-level architecture

**Figure 1:**
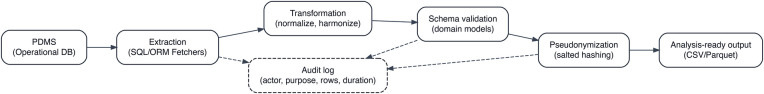
High-level data flow from PDMS read-only replica through extraction modules, schema validation, pseudonymization, and auditable export.

Data is retrieved from a read-only PDMS database replica using SQL- or ORM-based fetchers, transformed into standardized intermediate representations, validated against predefined schemas, and pseudonymized before export as analysis-ready CSV or Parquet files. In parallel, audit-log records are generated for key processing steps to document actor, purpose, row counts, and runtime, thereby supporting traceable and governance-compliant secondary use of PDMS data (Figure 1).

### 2.2 Data extraction

#### 2.2.1 Technical access

**Figure 2:**
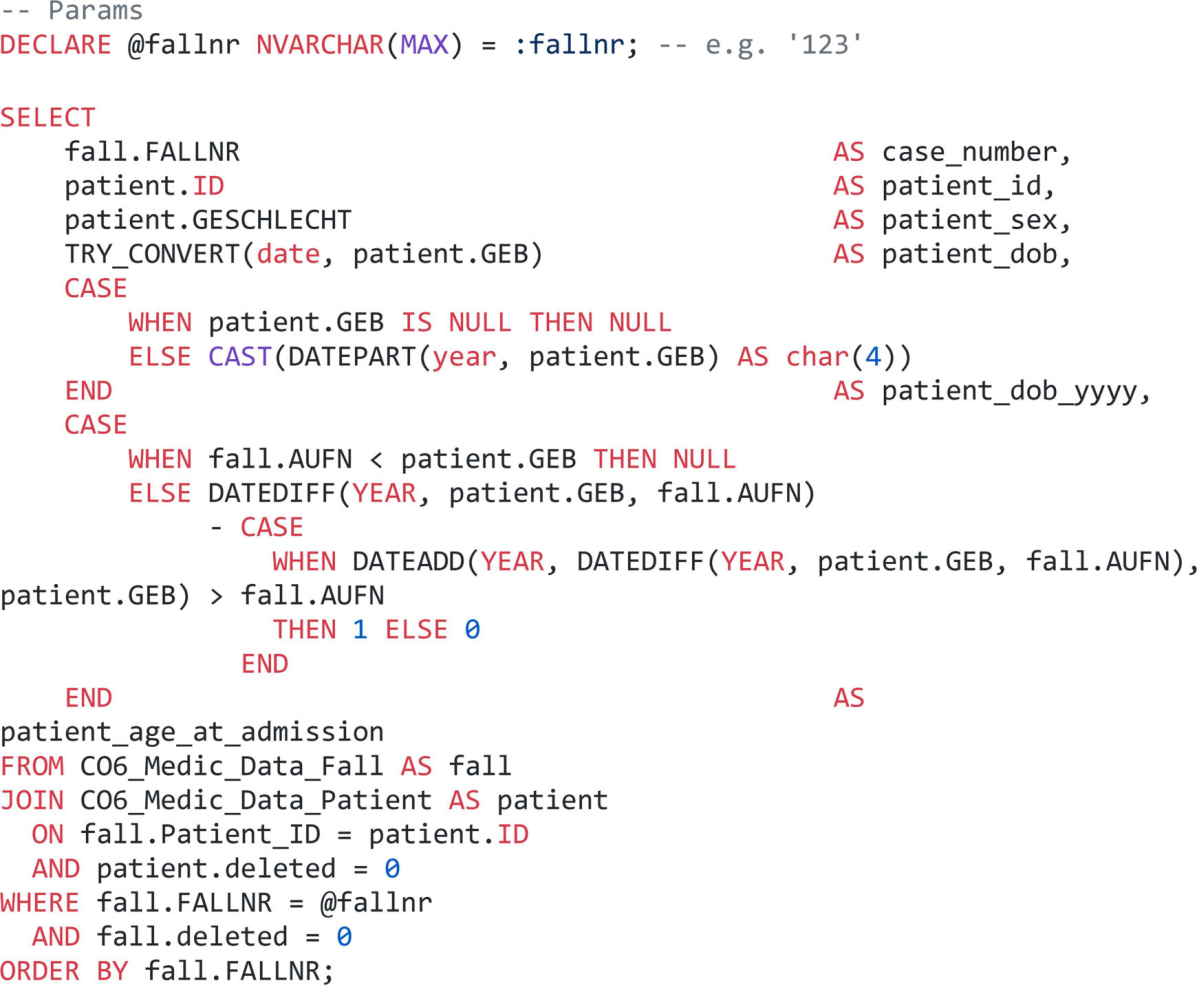
Straightforward query design for demographic data retrieval (SQL).

To minimize the risk of affecting clinical system performance, data access was established via a secured ODBC connection to a redundant, read-only copy of the operational database. Initial extractions utilized direct Structured Query Language (SQL) (10). A basic application is shown in Figure 2.

**Figure 3:**
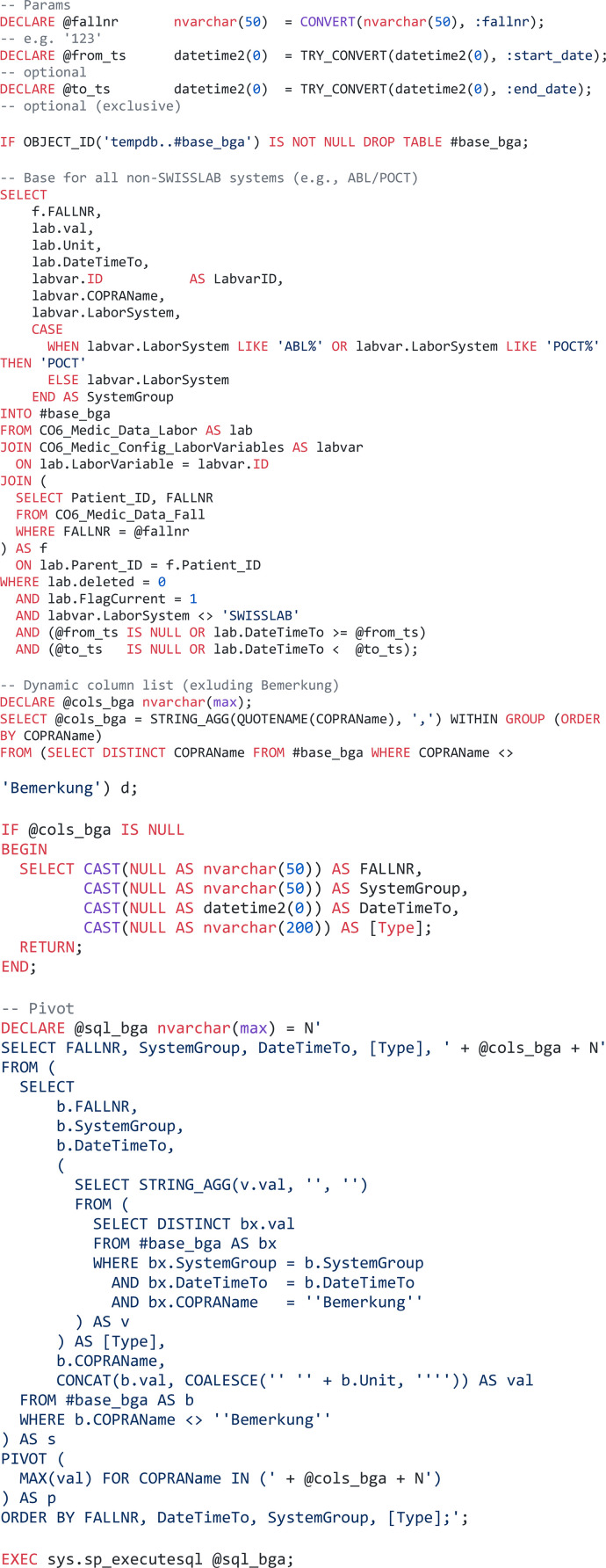
Complex query design for relational data structures and content-aware filtering, e.g. in point-of-care diagnostic applications (SQL).

Other, more relational data structures required a more complex extraction algorithm to ensure accurate outputs. Here, we describe the extraction of point-of-care blood gas analysis results (Figure 3).

As research demands expanded, a dedicated Python-based extraction framework was developed, using SQLAlchemy as an abstraction layer (11). This framework enabled reusable workflows across data domains (vital signs, therapies, medications), automated preprocessing steps such as time stamp conversion (UTC to CET/CEST), and implemented clinically necessary logic to address inconsistencies in documentation.

For example, documentation of device usage or therapy intervals often lacked explicit termination markers (end times). Furthermore, reverification of ongoing therapies always produced additional entries for the same treatment, resulting in multiple records for a single continuous intervention. Identifying truly ongoing interventions vs. documentation gaps was therefore critical. To address this, an interval-based logic was introduced that heuristically cross-referenced therapy records with patient bed occupancy data to infer missing end times and to rejoin fragmented therapy intervals into continuous episodes.

This modular pipeline facilitated reproducibility, improved transparency, and reduced errors compared with *ad hoc* SQL queries. To comply with legal requirements, the framework included an auditing mechanism for user-specific request logging and an integrated hashing for sensitive parameters.

### 2.3 Data transformation and harmonization

Extracted PDMS data required systematic data cleaning and restructuring. A modular transformation framework was implemented in Python, wherein each clinical domain (vital signs, medications, laboratory values, devices, demographics) was processed by a dedicated service module. Pydantic models defined the output, enforcing type safety, plausibility checks, and schema consistency.

#### 2.3.1 Temporal processing

All data streams were normalized to Central European Time (CET/CEST). For event-based records such as laboratory results or medication administrations, only the time stamps were converted. No additional aggregation or alignment was applied at this stage.

#### 2.3.2 Vital signs

Most numeric values (e.g., blood pressure, heart rate, ventilator parameters) were consistently stored with predefined units in the PDMS. Consequently minimal harmonization was required beyond plausibility filtering and time standardization.

#### 2.3.3 Medications

Medication data required the most extensive processing. Documentation occurred in various formats (volume, dose, or rate), and drug concentrations were often not explicitly available. Concentrations were therefore reconstructed from ingredient definitions using internal reference catalogs and classification algorithms. This step was essential to derive standardized application rates (e.g., mg/h, U/h) across projects.

#### 2.3.4 Devices and therapies

Support devices such as extracorporeal membrane oxygenation (ECMO), dialysis, microaxial pumps (e.g., Impella systems), or intra-aortic balloon pump (IABP) were documented as intervals, but end times were frequently missing. To resolve this, therapy records were cross-referenced with patient bed occupancy and related device tables, allowing inference of active vs. terminated therapies.

#### 2.3.5 Internal catalogs and variable definitions

Since international terminologies (e.g., LOINC, ATC) were unavailable in the source system, harmonization relied on internally maintained catalogs. These provided stable, project-wide definitions for drugs, laboratory parameters, and device attributes, serving as the central reference for variable identifiers to ensure consistency across service modules.

#### 2.3.6 Output and validation

Each service produced a validated, patient-centered output model. Primary validation via pydantic enforced schema conformity, rejected clinically implausible values, and ensured consistency. Final outputs were stored in structured, analysis-ready formats (CSV or Parquet) with standardized variable naming. Secondary validation involved a sample-based auditing process, comparing extracted data against values displayed in the clinical PDMS front end to confirm consistency.

#### 2.3.7 Usability and security

To make the extraction framework accessible beyond technically trained users, a lightweight front-end interface was implemented. The front-end interface incorporated fine-grained, user-based access control and audit logging, fulfilling institutional governance requirements as well as the traceability and accountability obligations defined under GDPR (Art. 5, 32) and Art. 27 BayKrG.

Predefined extraction templates for common research domains (e.g., epidemiology, vital parameters) provide standardized, validated data pipelines and minimize the risk of retrieving direct identifiers (e.g., patient name, date of birth). Extractions were tied to case numbers, enabling clinicians and researchers to obtain datasets relevant to specific trials or patient groups without requiring direct SQL interaction. Pseudonymization of sensitive identifiers was achieved by salted, irreversible hashing.

By abstracting low-level query complexity from end-user access, we reduced the risk of unintended database interference and promoted data integrity. In combination, the flexible back-end workflows and secure front-end interface created a scalable, reproducible, and governance-compliant process for secondary use of PDMS data.

Together, the secure front-end and modular back-end workflows provide auditable, role-restricted, and pseudonymized access to PDMS extracts in compliance with GDPR and Art. 27 BayKrG.

### 2.4 Data and code availability

A simplified version of the extraction framework demonstrating the demographic-data workflow is publicly available on GitHub (12). The full implementation in our hospital environment cannot be shared in its operational form, as extraction and transformation logic must be tailored to the institution-specific PDMS schema and is therefore neither directly portable nor readily interpretable outside our setting.

## 3 Results

### 3.1 Performance characteristics

To characterize operational performance, we measured throughput at two levels: * the framework's *processing layer* (schema validation, plausibility checks, and optional salted hashing), isolated from database access; and * *end-to-end extraction*, including database queries, domain-specific transformations, and validation. Unless noted otherwise, results summarize 5 repeated runs per cohort size, preceded by 1 warmup run. Only the repeated “run” phase is aggregated. To reduce dependence on a single fixed cohort and better reflect operational variability, case identifiers were **resampled per repeat** from a larger ID pool (seeded for reproducibility).

#### 3.1.1 Processing-layer throughput

Table 1 reports processing time for schema validation and optional hashing, excluding database query execution and network latency. These measurements isolate framework overhead from installation-specific factors.

**Table 1:** Processing-layer throughput for schema validation and optional salted hashing (single process).

Records	Runtime (s), no hashing	Throughput (rows/s), no hashing	Runtime (s), with hashing	Throughput (rows/s), with hashing
5,000	0.028	180,310	0.032	157,466
20,000	0.112	178,503	0.129	155,074
80,000	0.444	180,139	0.512	156,102

Throughput remained stable across record counts (∼180,000 rows/s without hashing, ∼156,000 rows/s with hashing), indicating near-linear scaling. Salted hashing adds approximately 13%–15% overhead—a predictable cost for irreversible pseudonymization.

#### 3.1.2 End-to-end extraction throughput

Table 2 reports end-to-end extraction performance including database query execution, domain-specific transformations, and validation. Demographics represents a simple extraction (one row per case); drugs represents a transformation-heavy extraction with medication parsing, concentration reconstruction, and interval processing.

**Table 2:** End-to-end extraction throughput for demographics (simple) and drugs (transformation-heavy) resources.

Resource	Cases	Records (mean)	Runtime (s)	Throughput (records/s)	Records/Case	Validation failures
Demographics	100	100.2	0.04	2,595	1.00	0%
Demographics	500	501.4	0.20	2,833	1.00	0%
Demographics	1,000	1,002.0	0.33	4,450	1.00	0%
Drugs	100	26,648.6	6.72	3,973	266.49	0%
Drugs	500	141,019.2	44.87	3,161	282.04	0%
Drugs	1,000	283,905.6	91.90	3,115	283.91	0%

For demographics, throughput was in the low-thousands of records per second and increased for larger cohorts after warmup, reflecting reduced one-time overheads (e.g., caching and initialization) in the aggregated runs. For the more complex drugs resource—requiring transformation-heavy event processing—throughput remained stable at approximately 3,100–4,000 records/s despite processing ∼266–284 medication events per case. Validation failure rates were 0% across all runs, indicating that the extracted records conformed to the defined schema contracts and plausibility checks in this sample.

#### 3.1.3 Multi-domain pipeline throughput

Table 3 reports performance for the complete multi-domain extraction pipeline **periop_study** (demographics, drugs, operating room timestamps, and aggregated drug windows).

**Table 3:** Multi-domain pipeline throughput for periop_study extraction (demographics + drugs + operating room timestamps + aggregations).

Cases	Total records (mean)	Runtime (s)	Throughput (records/s)
100	46,519.0	14.83	3,138
500	190,057.8	57.04	3,349
1,000	367,049.8	96.01	3,824

The near-linear scaling across case counts and the stable throughput (∼3,800 records/s at 1,000 cases) demonstrate that the framework handles complex, multi-domain extractions predictably. For larger cohorts, the extraction layer automatically chunks identifier lists into bounded batches to avoid database parameter limits and maintain stable query execution.

#### 3.1.4 Data quality observations

Benchmark runs also revealed upstream data quality characteristics in the sampled benchmark cohort: of 1,838 unique case numbers sampled, only 1 (0.05%) mapped to multiple patient identifiers, indicating rare but present identifier inconsistencies in the source system. No overlapping case admission windows were detected among 1,694 cases with complete timestamps, confirming temporal consistency in the source data for this sample.

### 3.2 Example usage of the pipeline for demographic data extraction, audit and de-identification

**Figure 4:**
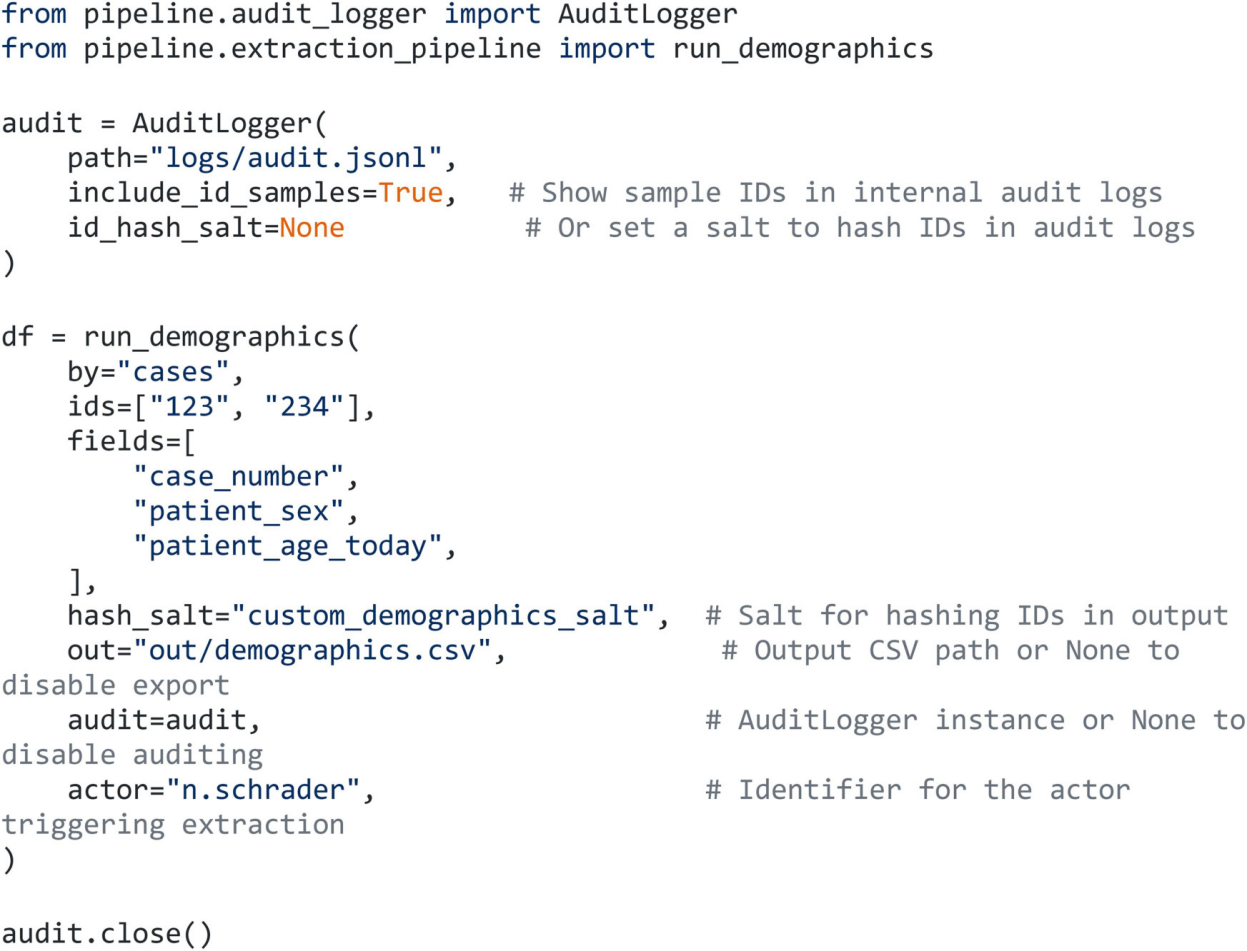
Application of the ETL pipeline to extract basic demographics, including audit logging and de-identification (Python).

To illustrate end-to-end behavior, Figure 4 shows an exemplary demographic extraction for two cases, including de-identification and audit logging.

**Figure 5:**
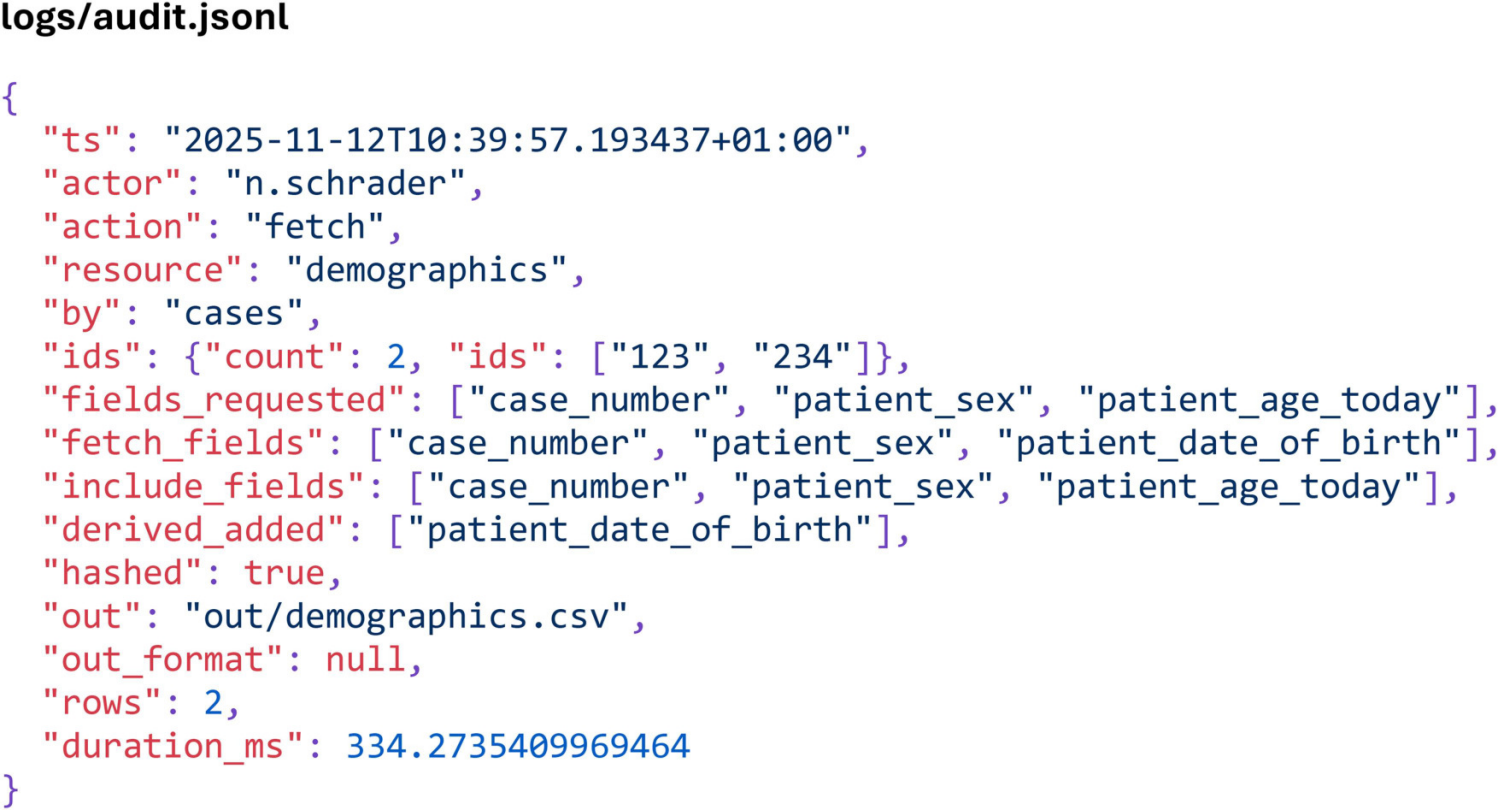
Output of the created audit log in JSON format.

**Figure 6:**
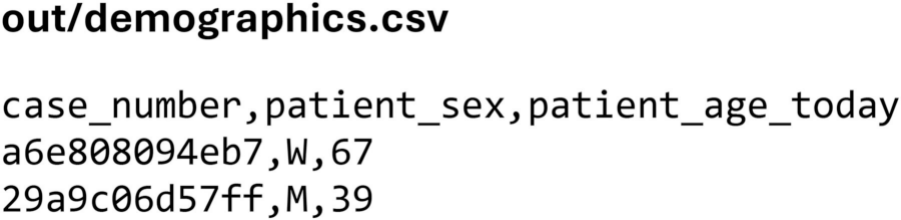
Output of the data extraction in comma-separated values format.

The corresponding audit log (Figure 5) and CSV output (Figure 6) document all parameters, derived fields, and pseudonymized identifiers, demonstrating transparent and traceable access to analysis-ready PDMS data.

## 4 Discussion

### 4.1 Technical and methodological considerations

The extraction of routine clinical data from PDMS presents fundamental technical and methodological hurdles. While optimized for flexible clinical documentation and transactional performance, these systems inherently lack robust tools for analytical data extraction. The highly fragmented, denormalized, and proprietary data structures necessitate highly complex, advanced queries. As a result, extensive schema knowledge will be necessary during the initial steps of data extraction. During these initial steps, the SQL-based approach is highly effective for targeted retrieval of domains (e.g., demographics, ventilator parameters, laboratory results). However, direct SQL data extraction lacked a robust mechanism for role-based access control beyond user-specific database credentials, and logging user-specific extraction requests was necessary to comply with national regulations.

Although de-identification of sensitive parameters via hashing in SQL would be technically feasible, and query-based parameter minimization is obvious, practical implementation and configuration for each individual use case proved highly complex. Furthermore, the use of custom units, varied time formats, and system-specific coding schemes requires extensive data harmonization. Consequently, without dedicated ETL pipelines, these rich datasets remain inaccessible to the majority of clinical researchers. All these hurdles have been described before (5, 13–15).

### 4.2 Component portability and reusability

Although query logic and mappings are necessarily PDMS- and site-specific, the framework was designed to separate reusable infrastructure from institution-specific adapters. Table 4 summarizes the portability of key components as reflected by the project structure.

**Table 4:** Portability characteristics of framework components. High portability indicates reuse without code changes; medium portability requires configuration or mapping; low portability is institution- or vendor-specific.

Component (repository layer)	Portability	Typical adaptation when porting
Database access abstraction (**connection/**)	High	Connection string/credentials/read-only replica
Time and hashing utilities (**helpers/datetime_helpers.py**, **helpers/hashing.py**)	High	Time zone policy, salt management, governance rules
Execution layer (registry, batching, export endpoints) (**pipeline/**)	High	Reuse as-is; register site-specific resources/adapters
Audit logging (**pipeline/audit_logger.py**)	High	Log sink, retention/rotation, ID sampling & hashing policy
Output contracts and plausibility checks (**schemas/**)	Medium	Extend/adjust schemas and plausibility rules per site
Local registries and catalogs (**constants/**)	Low	Update variable IDs, internal catalogs, mixture tables
PDMS schema models (**models/**)	Low	Replace with vendor-/site-specific ORM models
Domain extractors (**methods/**)	Low	Rewrite queries and joins for local PDMS schema

In practice, porting to a different PDMS installation requires updating the adapter layer (ORM models, domain-specific queries, and local registries), while leaving the core execution, validation, audit, and pseudonymization components unchanged.

### 4.3 Comparison with existing ETL approaches

The proposed framework targets governed extraction and transformation of high-frequency PDMS data into analysis-ready tables with explicit schema contracts and built-in pseudonymization and auditability. This focus differs from (i) pipelines built around public research datasets and (ii) common-data-model harmonisation projects. Table 5 summarizes these distinctions.

**Table 5:** Comparison of the proposed framework with established clinical ETL approaches.

Framework	Primary target	Standardization	Audit integration	Pseudonymization	Source access
OMOP CDM ETL tools	Multi-site research	OMOP vocabularies	Limited	External	Full schema required
FHIR exporters	Interoperability	FHIR resources	Variable	External	API-based
MIMIC-Extract	Research (public data)	Custom	None	Pre-applied	Public dataset
i2b2 ETL	Research networks	i2b2 ontology	Basic	External	Star schema
Proposed framework	Institutional	Internal catalogs	Integrated	Embedded	Direct database

OMOP, observational medical outcomes partnership; FHIR, fast healthcare interoperability resources; i2b2, informatics for integrating biology and the bedside.

MIMIC-Extract provides a well-known extraction and preprocessing pipeline for the MIMIC-III dataset, emphasising reproducible feature generation on a fixed, public ICU schema (16). In contrast, PDMS installations in routine care use proprietary schemas and local variable registries. Therefore, our approach emphasises a clear adapter layer for site-specific extraction combined with a portable core for validation, traceability, and governance.

Common-data-model ecosystems such as OHDSI/OMOP prioritise semantic interoperability and cross-site comparability, typically requiring extensive mapping to standard vocabularies and a CDM schema (17). Full semantic interoperability requires continuous vocabulary management, extensive mapping effort, and ongoing maintenance as PDMS configurations evolve. For many ICU research and quality-improvement use cases, the marginal benefit of immediate CDM conversion is outweighed by the operational cost and risk of introducing mapping errors. Our framework can serve as a preceding step by producing quality-controlled, pseudonymised exports that can then be mapped to a CDM when needed.

Finally, interoperability standards such as SMART on FHIR facilitate app-level integrations where FHIR interfaces are available (18). However, for many PDMS-backends, direct relational extraction remains necessary for high-frequency bedside signals and vendor-specific documentation structures.

### 4.4 Limitations and trade-offs

The framework does not aim to produce a fully standardized interoperability output (e.g., OMOP CDM or FHIR resources) directly from the PDMS. This is not an omission but a deliberate separation of concerns: PDMS databases are often proprietary and locally customized, and forcing full semantic harmonization at the point of extraction increases implementation complexity, reduces transparency, and slows iteration. Our approach therefore prioritizes traceability (audit logs), reproducibility (schema contracts), and usability (rapid integration by domain experts), while treating interoperability as an optional downstream transformation step.

In secondary-use contexts governed by GDPR, the ability to demonstrate who accessed which data, for what purpose, and how records were transformed is operationally critical. By embedding audit logging and pseudonymization directly into the extraction pipeline, the framework provides governance guarantees that many general-purpose ETL stacks or ad-hoc scripts do not. Standardization can still be applied later, but governance must be established at the point of data access.

Although the output is not a universal interoperability standard, each domain export follows a defined explicit schema contract with validation and plausibility checks. This provides a strong form of syntactic consistency across extractions and enables incremental harmonization (e.g., mapping selected domains to OMOP or FHIR profiles) without destabilizing the extraction layer.

A practical strength of the architecture is its low barrier to adoption. The modular design, explicit schema definitions, and auditable execution allow integration and maintenance by a single clinically trained developer with limited institutional overhead. This is often the only realistic pathway for initiating secondary-use pipelines in resource-constrained environments.

Future work will focus on optional mapping modules to standardize representations (e.g., OMOP/FHIR) for selected domains, leveraging the existing schema contracts and audit trail to keep transformations traceable.

### 4.5 Ethical and legal considerations

All data handling complied with the European General Data Protection Regulation (GDPR; German: Datenschutz-Grundverordnung, DSGVO). The processing of special categories of personal data for scientific research is permitted under Art. 9(2)(j) GDPR in conjunction with appropriate safeguards, while the general conditions for lawful processing are governed by Art. 6(1) GDPR. In Bavaria, the specific legal basis enabling hospital-internal research with pseudonymized routine data is Art. 27 Bayerisches Krankenhausgesetz (BayKrG), which permits the secondary use of hospital data for scientific purposes without individual patient consent, provided that suitable technical and organizational measures are implemented. These provisions are complemented by the Bundesdatenschutzgesetz (BDSG) and, in a more general manner, the Bayerisches Datenschutzgesetz (BayDSG), which establish additional requirements for processing personal data in public institutions. At the federal level, the Gesundheitsdatennutzungsgesetz (GDNG) introduces further provisions for the use of health data in research and quality-improvement contexts, thereby reinforcing the legal framework established by the DSGVO and BayKrG.

In line with Art. 32 GDPR and the security requirements defined in these laws, state-of-the-art technical and organizational measures were applied to ensure confidentiality, integrity, and availability of the data. Patient identifiers were removed at the time of extraction and replaced with salted, irreversible pseudonyms. All data linkage across domains was conducted exclusively within this pseudonymized environment.

Access to raw datasets was limited to authorized staff bound by professional confidentiality and institutional governance rules. All extraction and transformation steps were performed within a secure computing environment physically located at the university hospital, with no transfer of personal data to external servers or cloud infrastructure. Audit logs documented every data access and pipeline execution, ensuring full traceability.

## 5 Conclusion

In conclusion, routine data stored in PDMS contain unique opportunities for clinical and translational research, and the development of automation and decision-support systems. However, secondary use requires overcoming substantial technical and methodological barriers. The approach presented here offers a reproducible methodology for extracting, transforming, harmonizing, and validating PDMS data, making them accessible to a wider research community while maintaining compliance with ethical and legal requirements. By prioritizing governance, auditability, and schema contracts over premature standardization, the framework provides a practical foundation upon which interoperability efforts can be built incrementally.

## Data Availability

The datasets presented in this study can be found in online repositories. The names of the repository/repositories and accession number(s) can be found below: https://github.com/DataScienceUKW/pdms_data_processing_pipeline.
